# Optimization of Methodology for Simultaneous Quantification of Trigonelline, 5‑Caffeoylquinic Acid, and Caffeine in Green and Roasted Coffee Extracts by HPLC

**DOI:** 10.1021/acsomega.5c05526

**Published:** 2025-08-25

**Authors:** Walace Breno da Silva, Larissa Martins Rocha, Lucca Dornelas Guimarães Moura, Márcio Santos Soares, Sabrina Alves da Silva, Daniele Birck Moreira, Pedro Ivo Vieira Good God, Geraldo Humberto Silva

**Affiliations:** † Institute of Exact and Technological Sciences, Laboratory of Development of Natural Agrochemicals, 635369 Federal University of Viçosa, Highway MG-230, Km 7 - Rural Area, Rio Paranaíba 38810-000, MG, Brazil; ‡ Institute of Agrarian Sciences, Laboratory of Biochemistry and Molecular Genetics, Federal University of Viçosa, Highway MG-230, Km 7 - Rural Area, Rio Paranaíba 38810-000, MG, Brazil; § Institute of Exact and Technological Sciences, Laboratory of Environmental Geochemistry and Natural Products, Federal University of Viçosa, Highway MG-230, Km 7 - Rural Area, Rio Paranaíba 38810-000, MG, Brazil; ∥ Institute of Animal Health and Production, 89116 Federal Rural University of the Amazon, Avenue Presidente Tancredo Neves, N° 2501, Terra firme, Belém 66077-830, PA, Brazil; ⊥ Laboratory of Molecular Genetics of Plants and Phytopathogens, 67845 Mato Grosso State University Pro-Central-West Network, Highway MT-358, Km 7, Jardim Aeroporto, Tangará da Serra 78301-532, MT, Brazil

## Abstract

The main bioactive compounds in coffee beans affecting beverage quality include trigonelline, 5-caffeoylquinic acid (5-CQA), and caffeine. Despite the use of various analytical techniques, there is a need for a faster and more accessible alternative to the costly and complex methods used in routine coffee composition analysis. This study aimed to optimize and validate a high-performance liquid chromatography with ultraviolet detection (HPLC-UV-DAD) method for the simultaneous quantification of these three bioactive compounds in five *Coffea arabica* cultivars (Yellow Catuai, Guesha, Arara, Red Catuai, and Laurina). The evaluated parameters included linearity, accuracy, robustness, precision, limit of quantification, and detection. The proposed method met the criteria of the analytical validation figures of merit, being considered to be fast and effective for determining these compounds. In addition, the study of the concentration of these compounds in the different coffee cultivars showed significant differences, with the Laurina cultivar having the lowest concentration of caffeine (7.9 mg g^–1^). Between raw and roasted beans, there were significant variations in the degradation rates of 5-CQA and trigonelline, with the highest for 5-CQA in the Arara cultivar (62.59%) and the highest for trigonelline (28.76%) in the Laurina cultivar. The speed and simplicity of the method can be used to investigate the contribution of these bioactives to the sensory characteristics of the drink and their possible health benefits.

## Graphical abstract

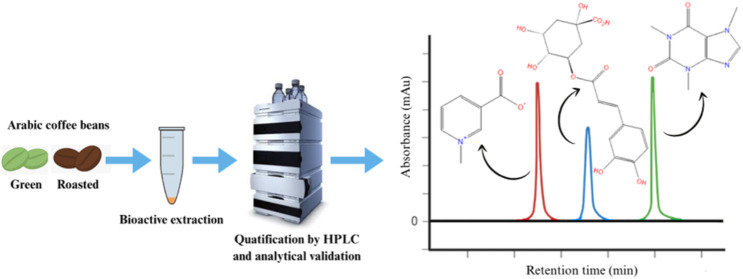

## 1 Introduction

The coffee bean has a complex chemical composition, with more than 800 volatile and nonvolatile compounds identified in the different species and cultivars. This great diversity of substances is also determined by the soil and climatic conditions in which the coffee is grown as well as the different methods of cultivation, harvesting, drying, and roasting used. Various studies have been carried out to understand the functions of compounds or classes of substances that influence the quality of the drink and human health.^1^ Several compounds in the drink are considered bioactive, as they confer health benefits due to their antioxidant and anti-inflammatory properties and influence the drink’s quality.^2^ Among the various bioactive compounds, trigonelline, 5-caffeoylquinic acid, and caffeine stand out.

Trigonelline, an alkaloid compound, can neutralize free radicals in the body, which helps protect cells from oxidative damage. During the roasting process, it can be degraded to niacin, known as vitamin B3, which studies have shown is effective in lowering cholesterol and acts on specific receptors to reduce the release of fatty acids from adipose tissue.^3,4^ Trigonelline impacts beverage quality, as its degradation produces compounds such as pyrazines and pyrroles, which are associated with various odors such as earthy, almond, burnt, green, and nutty.^5^

5-Caffeoylquinic acid (5-CQA), an ester formed between caffeic acid and quinic acid, is one of the chlorogenic acids with an essential role in determining the acidity and complexity of the beverage’s taste.^6^. This compound has antioxidant and anti-inflammatory properties that may prevent chronic diseases.^7^ This phenolic acid has significant antioxidant properties and has been shown to have anti-inflammatory effects by reducing inflammation in some pathological conditions.^8^ In vitro and in vivo studies have suggested that 5-CQA may protect against various chronic diseases, such as cardiovascular disease, by modulating metabolic and inflammatory pathways. Recent studies indicate that 5-CQA modulates glucose metabolism by intervening in insulin sensitivity and glucose absorption, which could be used to treat type 2 diabetes.^9–11^

Caffeine is the best-known compound in coffee and belongs to the class of compounds known as xanthines, specifically methylxanthines.^12^ It has stimulating properties on the central nervous system, so it is recommended to use it in small doses at night.^13^ Several studies have been conducted to verify the benefits of caffeine for human health. Studies have shown that daily caffeine consumption in men reduces the risk of developing Parkinson’s disease by at least 5 times. However, the same effect is not observed in women taking estrogens in postmenopausal hormone replacement therapy, suggesting that caffeine interacts with estrogens.^14^ Caffeine also plays a crucial role in the drink’s bitterness,^15^ justifying the importance of decaffeinated coffee production for the coffee economy.

Various analytical techniques have been employed in the characterization of bioactive compounds and in the authentication of coffee, including ultraviolet (UV) spectroscopy and liquid chromatography coupled with mass spectrometry (LC-MS).^16^ Recent studies have demonstrated the feasibility of quantifying compounds such as sucrose, caffeine, and trigonelline in green coffee beans using advanced techniques such as hyperspectral imaging (HSI), although these approaches require sophisticated instrumentation and involve higher analytical complexity.^17^ Simplified methodologies have been proposed for the determination of alkaloids and phenolic acids in coffee, such as the use of the QuEChERS method coupled with ultraviolet–visible (UV–vis) spectrophotometry for detecting trigonelline, caffeine, and 5-caffeoylquinic acid in green coffee bean extracts.^18^ However, this approach presents limitations, as the spectral signals of caffeine and chlorogenic acids overlap in the 200–500 nm range, hindering their simultaneous quantification in simple aqueous extracts.^19^ Therefore, UV–vis spectroscopy lacks adequate specificity in complex matrices, such as coffee infusions.

From a chromatographic standpoint, HPLC-based methodologies have proven to be effective for the simultaneous quantification of these compounds. For instance, Santiago et al. (2020) developed a method for the simultaneous analysis of trigonelline, caffeine, and 5-caffeoylquinic acid with a total chromatographic runtime of 20 min.^20^ Additionally, Mehari et al. (2015) reported the application of HPLC in the quantification of alkaloids in green coffee beans, including trigonelline, caffeine, theobromine, and theophylline. However, due to the naturally low concentrations of theobromine and theophylline in coffee beans, the methodology has limitations in simultaneously quantifying all target components in the coffee matrix.^21^

Nevertheless, these methods often require elaborate sample preparation steps, such as extractions with organic solvents and filtration, which negatively impact their applicability in routine analyses and their sustainability profile. In this context, this study proposes a more straightforward and sustainable approach for the simultaneous analysis of trigonelline, 5-caffeoylquinic acid, and caffeine in extracts from both raw and roasted coffee beans. This method is based solely on the direct infusion of ground coffee beans with hot water, eliminating the use of organic solvents and reducing the sample preparation time. The analysis is performed using high-performance liquid chromatography with a diode array UV detector (HPLC-UV-DAD), with a short analysis time. Accurate determination of these compounds contributes to a better understanding of the chemical and sensory characteristics of coffee, facilitating the standardization and optimization of postharvest processes to enhance and ensure product quality, thereby adding value. Furthermore, proper method validation increases the reliability of results, enabling comparisons across different studies and laboratories.^22^

## 2 Materials and Methods

### 2.1 Standards and Reagents

Acetonitrile (Sigma-Aldrich, St Louis) HPLC grade 99.9%, glacial acetic acid (Fmaia, São Paulo, Brazil) UV/HPLC 99.7%, ultrapure water obtained by the Milli-Q-Plus system (Millipore Corporation, Darmstadt, Germany). Trigonelline standards (Sigma-Aldrich, St Louis) were 98.5% pure, 5-caffeoylquinic acid (Sigma-Aldrich, St Louis) 95% purity, and caffeine (Sigma-Aldrich, St Louis) 99% purity, all in the solid state Figure 1.

**Figure 1:**
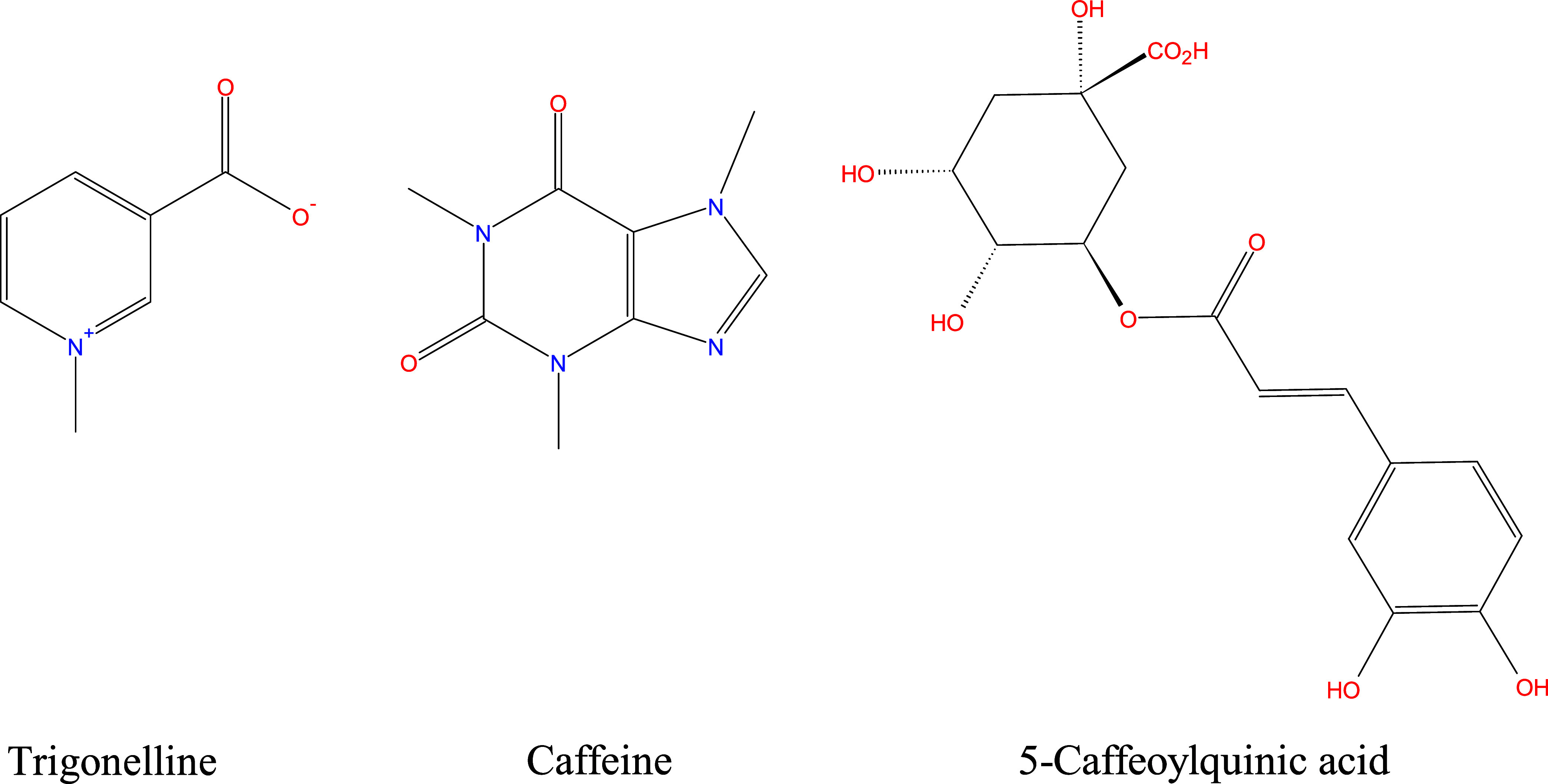
Structures of the bioactive compounds used in the validation of the methodology.

### 2.2 Equipment

An Agilent 1260 HPLC liquid chromatograph equipped with a G7111B 1260 Quat pump, a G7129A 1260 Vial sampler autosampler, and a G7117C 1260 DAD HS detector were used (Agilent, Santa Clara). The column used was a Luna Omega Polar C18 LC column with an internal diameter of 4.6 mm, length of 150 mm, and particle size of 5 μm (Phenomenex, Aschaffenburg, Germany) connected to an Eclipse XDB-C18 precolumn with an internal diameter of 4.6 mm, length 12.5 mm, particle size 5 μm (Agilent, Santa Clara).

### 2.3 Coffee Samples

Three replicates of raw and roasted beans from 5 cultivars harvested in the 2023 crop in the Alto Paranaba region (Minas Gerais State, Brazil) were used. The beans were subjected to the same roasting process but processed using different postharvest methods (Table 1). Unpeeled cherry fruits were used for all postharvest methods.

**Table 1:** Coffee Cultivars and Their Respective Postharvest Processing Methods Used for Bioactive Compound Quantification

cultivars	method of postharvest
Yellow Catuai	carbonic maceration
Guesha	carbonic maceration
Red Catuai	natural
Arara	aerobic fermentation
Laurina	natural

### 2.4 Sample Preparation

The raw and roasted beans were ground in a cryogenic mill (IKA A11basic S032) using liquid nitrogen to facilitate grinding. After grinding, the samples were passed through a 20-mesh stainless steel 304 sieve to standardize the particle size. After this process, the samples were sent to HPLC-UV for analysis.

### 2.5 Figures of Merit Used in the Validation of the Analytical Method by HPLC-UV

The method for the simultaneous analysis of trigonelline, 5-caffeoylquinic acid (5-CQA), and caffeine in raw and roasted coffee extracts by HPLC-UV was validated. The following parameters were analyzed: linearity, accuracy (recovery), precision (reproducibility), robustness, limit of quantification (LOQ), and limit of detection (LOD).

#### 2.5.1 Linearity

The construction of the analytical curves determined the linearity of the method. Standard solutions were prepared in ultrapure water by solubilizing the respective reference substances (trigonelline, 5-caffeoylquinic acid, and caffeine). Six subsequent solutions were prepared at concentrations of 1.0, 2.0, 4.0, 10.0, 20.0, 30.0, 40.0, 50.0, and 60.0 μg mL^–1^ for each compound in triplicate on consecutive days to minimize errors due to external conditions such as temperature, humidity, etc. The analytical curve was obtained by linear regression, and the quality of the results was analyzed using variance correlation and determination coefficients.

#### 2.5.2 Accuracy

The recovery test assessed accuracy by adding the standards to coffee samples with known concentrations of the analytes (in raw coffee—trigonelline: 6.67 μg mL^–1^; 5-CQA: 15.34 μg mL^–1^; caffeine: 8.34 μg mL^–1^ and in roasted coffee— trigonelline: 13.34 μg mL^–1^; 5-CQA: 10.67 μg mL^–1^; caffeine: 16.67 μg mL^–1^). The following concentrations of the bioactive compounds were added: 20.0, 30.0, 40.0, 50.0, and 60.0 μg mL^–1^.

#### 2.5.3 Precision

Precision was assessed through reproducibility and analyzed in quintuplicate and on three alternate days. The study used standard solutions at 5, 15, 35, and 55 μg mL^–1^ concentrations. The results were expressed by the dispersion of the results and calculating the relative standard deviation (RSD) of the series of measurements.

#### 2.5.4 Robustness

Robustness was planned using the Plackett-Burman method to check the effect of multiple variations (Table 2) on the chromatographic method.^23^ The seven factors were evaluated, considering the nominal condition and the variation at a higher level, and combined in eight tests. The analyses were carried out using standard solutions of the analytes. The data were analyzed using the Student’s *t* test of Lenth’s method at a significance level of *p* < 0.05, considering the variation in retention time and area.

**Table 2:** Parameters and Variations Were Used in the Plackett-Burman Experimental Design to Assess Robustness^a^

	conditions		combination of factors							
parameters	nominal (1)	variation (−1)	1	2	3	4	5	6	7	8
p1	15	16	–1	–1	–1	1	–1	1	1	1
p2	40	42	1	–1	1	1	–1	–1	–1	1
p3	1	1.1	–1	1	1	–1	–1	–1	1	1
p4	272	274	–1	1	–1	1	1	–1	–1	1
p5	Êxodo Científica	Dinâmica Química Contemporânea LTDA	1	–1	–1	–1	1	–1	1	1
p6	1	1.5	–1	–1	1	–1	1	1	–1	1
p7	acetic	formic	1	1	–1	–1	–1	1	–1	1

a (p1) Acetonitrile concentration in the mobile phase; (p2) column temperature (°C); (p3) mobile phase flow rate (mL/min); (p4) wavelength (nm); (p5) acetonitrile brand; (p6) acid concentration in the mobile phase; and (p7) acid type.

#### 2.5.5 Limit of Quantification

The limit of quantification was determined from the lowest value of the calibration curve used in the linearity test. The signal-to-noise ratio was also analyzed using OpenLab software (Agilent LC 1260) to verify a value greater than 10:1.

#### 2.5.6 Limit of Detection

The detection limit was estimated using the Agilent LC 1260 OpenLab software based on a signal/noise ratio greater than 3:1. Three replicates of the blank were analyzed on each day of the method′s validation.

#### 2.5.7 Matrix Effect

Three calibration curves were constructed using different concentrations of trigonelline, 5-caffeoylquinic acid, and caffeine: one based on the linearity of the method at concentrations of 20.0, 30.0, 40.0, 50.0, and 60.0 μg mL^–1^, the other two considering the addition of analyte standard (doping) at the same concentrations in the raw and roasted coffee extracts. The relationship between the area obtained and the concentration was analyzed by linear regression, and the angular and linear coefficients were compared by coincidence and intercept tests.

### 2.6 Analysis by HPLC-UV

The analysis method was adapted from da Silva.^24^ The chromatographic condition used was the gradient mode, with the mobile phase consisting of a solution of water with 1% acetic acid (solvent A) and acetonitrile (solvent B), following the following proportion: 85% solvent A and 15% solvent B for 5 min and column cleaning and 75:25 v/v for another 10 min, with the detector at a wavelength of 272 nm, an oven temperature of 40 °C, an injection volume of 10 μL and a flow rate of 1.0 mL min^–1^.

### 2.7 Extraction of Bioactive Compounds

The bioactive compounds were extracted using the methodology adapted from Duarte et al.,^25^ where 0.5 g of the samples was poured into 50 mL of distilled water were placed in a water bath at 90 °C for 5 min. The mixture was filtered through no. 4 filter paper and a 0.45 mm hydrophilic syringe filter. The filtrate was diluted 16 times for raw coffee and 8 times for roasted coffee.

### 2.8 Software and Statistical Analysis

The figures of merit were analyzed using the OpenLab software (Agilent LC 1260) for the chromatograph and *Action Stat* for Windows.^26^ Statistical analysis was carried out to compare the means between the cultivars and the raw and roasted process using the *SPEED Stat* software,^27^ using the Tukey test with a significance level of 5%.

## 3 Results and Discussion

### 3.1 Method Validation

To check the quality and resolution of the chromatograms of the raw and roasted coffee extracts, the standard was added to the raw and roasted extracts, and the same concentration was added to both extracts (10 μg mL^–1^) in triplicate. An elution time of less than 5 min was observed for the analytes and good resolution of the chromatographic bands (Figure 2).

**Figure 2:**
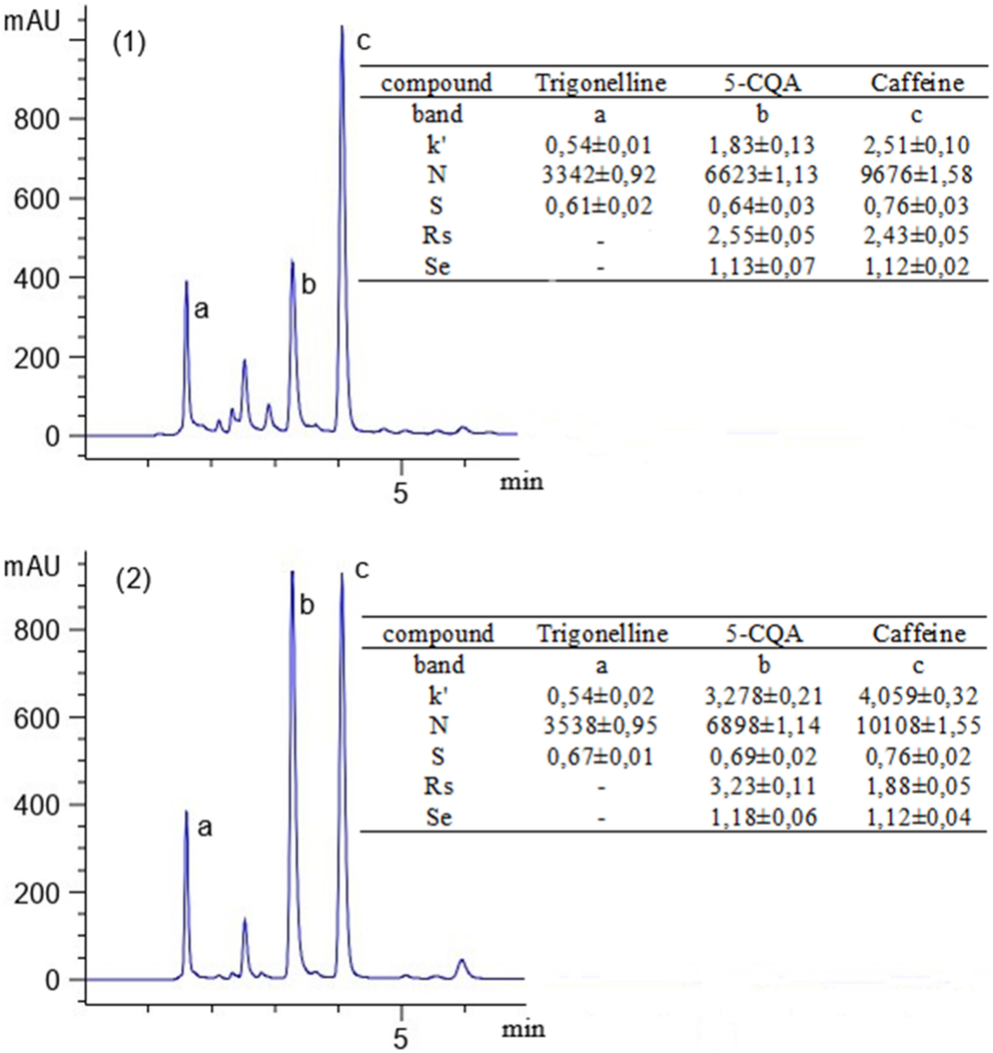
Chromatogram with the analytes trigonelline (band a), 5-CQA (band b), and caffeine (band c). Conditions: Luna Omega Polar C18 column (internal diameter 4.6 mm, length 150 mm, particle size 5 μm); mobile phase: water with 1% acetic acid: acetonitrile (85:15); flow rate 1 mL min^–1^; detection: UV 272 nm, roasted coffee (1) and raw coffee (2), *k*’ is the retention factor; *N* is the number of theoretical plates; *S* is the symmetry; Rs is the resolution, and Se is the selectivity.

The results for the theoretical plates were above the acceptance criteria of the Food and Drug Administration,^28^ which states that they should be higher than 2000. The symmetry of the bands was above 0.65, which indicates that the characteristics of the compounds influence the symmetry. Better symmetry can be obtained by changing the chromatographic conditions, such as column pressure and mobile phase flow rate.^29^ Despite the symmetry values being higher than 0.65, the evaluated accuracy and precision demonstrate that the method is reliable. Trigonelline had a *k’* of less than 1, which shows its low affinity with the stationary phase. Resolution and selectivity were also affected by trigonelline’s poor interaction with the stationary phase, which was not the case with 5-CQA and caffeine.

#### 3.1.1 Linearity Results

The data obtained for constructing analytical curves showed a homoscedastic behavior. When submitted to the Cochran test, the calculated C values for the three analytes were lower than the tabulated value (0.616), so the curve was constructed using ordinary least-squares. The correlation and regression coefficients were higher than 0.990, which indicates adequate linear behavior (Table 3).

**Table 3:** Regression Statistics for the Linearity Analysis of Trigonelline, 5-Caffeoylquinic Acid, and Caffeine

	trigonelline	5-caffeoylquinic acid	caffeine
correlation coefficient	0.9996	0.9979	0.9997
*R* ^2^	0.9997	0.9995	0.9998
linear coefficient	126.680	–18.600	316.980
angular coefficient	71.053	59,371	189.380
limit of quantification (μg/mL)	1.000	1.000	1.000
limit of detection (μg/mL)	0.450	0.450	0.450
C calculated	0.573	0.487	0.278

The quality of the linear regression was assessed by the ANOVA F test, where the angular coefficient for all analytes was significant (*p*-value <0.001), meaning that *y* varies as a function of *x*, and the angular coefficient is different from zero (Table S1, Supporting Information). For trigonelline and caffeine, the calculated *t* values were greater than the tabulated *t* values (2.12). Therefore, the linear coefficient was assumed to be different from zero, and for 5-CQA, the calculated *t*-value (−2.58) was smaller than the tabulated one. Therefore, it was necessary to analyze the *p*-value result, which was 0.0123. Thu,s it was assumed that the intercept differed from zero (Table S2, Supporting Information).

#### 3.1.2 Accuracy Results

The average recovery values (Table 4) in the roasted coffee extract samples showed a minimum value of 90.4% for caffeine and a maximum value of 97.8% for 5-caffeoylquinic acid. It should be noted that the values did not exceed 100%, which indicates a slight deviation in the direction of decreasing levels. However, the values did not exceed those permitted according to INMETRO guidelines (90–107%).^30^

**Table 4:** Average Recovery Values of Bioactive Compounds in Roasted Coffee Extracts

compound	trigonelline		5-cafeoylquinic acid		caffeine	
theoretical concentration (μg mL^–1^)	experimental concentration (μg mL^–1^)	recovery (%)	experimental concentration (μg mL^–1^)	recovery (%)	experimental concentration (μg mL^–1^)	recovery (%)
20	19.4 ± 0.5	97.0	18.1 ± 0.9	90.5	18.5 ± 0.4	92.5
30	27.7 ± 0.8	92.3	29.2 ± 1.1	97.3	27.1 ± 0.8	90.4
40	37.2 ± 1.1	93.0	37.8 ± 1.3	94.5	38.7 ± 1.1	96.7
50	48.1 ± 1.5	96.2	48.2 ± 1.7	96.4	46.4 ± 1.5	92.8
60	57.0 ± 2.0	95.0	58.7 ± 2.1	97.8	56.8 ± 1.9	94.7

The average recovery values in the raw coffee extract samples (Table 5) showed a maximum value of 112.5% for the lowest trigonelline concentration and a minimum value of 96.6% for the lowest caffeine concentration. It can be seen that the only value that exceeded 10% of the theoretical concentration added refers to the lowest concentration of trigonelline.

**Table 5:** Average Recovery Values of Bioactive Compounds in Raw Coffee Extracts

compound	trigonelline		5-cafeoylquinic acid		caffeine	
theoretical concentration (μg mL^–1^)	experimental concentration (μg mL^–1^)	recovery (%)	experimental concentration (μg mL^–1^)	recovery (%)	experimental concentration (μg mL^–1^)	recovery (%)
20	22.5 ± 0.4	112.5	21.7 ± 0.4	108.5	19.32 ± 0.3	96.6
30	32.2 ± 0.7	107.3	32.2 ± 0.7	107.3	29.1 ± 0.6	97.0
40	43.2 ± 0.9	108.0	42.8 ± 0.9	107.0	38.6 ± 0.8	96.5
50	53.1 ± 1.2	106.2	53.5 ± 1.1	107.0	48.5 ± 1.0	97.0
60	64.6 ± 1.5	107.7	63.6 ± 1.6	106.0	59.1 ± 1.2	98.5

According to Resolution of the Collegiate Board of Directors (RDC) No. 166 of July 24, 2017, of the National Health Surveillance Agency (ANVISA),^31^ there is no defined recovery percentage value to assess the method’s accuracy. Still, since the data were not highly variable, the recovery showed good values.

#### 3.1.3 Precision Results

The accuracy assessment carried out by the method’s reproducibility test showed that the relative standard deviation (RSD) values for trigonelline ranged from 0.15 to 2.91%, 0.54 to 3.30% for 5-caffeoylquinic acid, and 0.30 to 3.85% for caffeine. These values are lower than 5.0%, as indicated by ANVISA for suitable method accuracy Table 6.

**Table 6:** Values for the Evaluation of the Accuracy of the Chromatographic Method in the Simultaneous Determination of Trigonelline, 5-Caffeoylquinic Acid, and Caffeine

compound	trigonelline		5-cafeoylquinic acid		caffeine	
theoretical concentration (μg mL^–1^)	experimental concentration (μg mL^–1^)	relative standard deviation (%)	experimental concentration (μg mL^–1^)	relative standard deviation (%)	experimental concentration (μg mL^–1^)	relative standard deviation (%)
5	5.11	2.91	5.38	3.30	5.38	3.85
15	15.46	2.05	15.17	0.95	15.58	1.69
35	35.44	0.15	35.32	0.73	35.74	1.03
55	55.35	0.58	55.23	0.54	56.10	0.30

#### 3.1.4 Robustness Results

Using Lenth’s *t*-Student test (Table 7), only the wavelength, considering the retention time, for 5-caffeoylquinic acid showed a significant effect (p-value 0.0317); the effect value was 0.4914 and was above the value of 0.3964, the margin of error (ME) (Figure S1, Supporting Information). Regarding the area, the effect of the wavelength was significant only for caffeine (*p*-value 0.0127), with a value of 0.6244, higher than the ME of 0.3335 (Figure S2, Supporting Information). The method’s robustness is, therefore, considered good, except for the wavelength variation in the two compounds.

**Table 7:** Effect of the Variations Obtained by Plackett-Burman Planning and Student-*t* Test^a^

	trigonelline		5-cafeoylquinic acid		caffeine	
parameters	*p*-value for RT	*p*-value for area	*p*-value for RT	*p*-value for area	*p*-value for RT	*p*-value for area
*p*1	0.5649	0.4123	0.6491	0.1809	0.0571	0.2916
*p*2	0.5649	0.5649	0.1463	0.8895	0.7913	0.8332
*p*3	0.1937	0.9437	0.5649	0.3543	0.9468	0.6461
*p*4	0.3257	0.2626	**0.0317**	0.8306	0.1991	**0.0127**
*p*5	0.3910	0.5876	0.2777	0.5649	0.6491	0.1108
*p*6	0.7985	0.9251	0.5649	0.5758	0.2124	0.7465
*p*7	0.5649	0.4881	0.7421	0.4048	0.4900	0.4924

a (*p*1) Acetonitrile concentration in the mobile phase; (*p*2) column temperature (°C); (*p*3) mobile phase flow rate (mL/min); (*p*4) wavelength (nm); (*p*5) acetonitrile brand; (*p*6) acid concentration in the mobile phase; (*p*7) acid type.

#### 3.1.5 Matrix Effect Results

The *p*-values >0.05 in the slope comparisons for the three analytes indicate no significant difference between the slopes, with the curves being parallel, which means that doping does not alter the method’s sensitivity. The *p*-values <0.05 indicate a statistical difference in the intercepts, demonstrating that the lines do not coincide. The Supporting Information shows the curves and the respective results of the intercept and slope tests in Figures S3, S4, and S5.

### 3.2 Application of the Method to Quantify Bioactive Compounds

Validation of the method showed that the results were within established standards, confirming its suitability for quantification of bioactive compounds in coffee. This quantification helps us to study and understand how these compounds influence the sensory characteristics of the beverage. The method was then applied to measure three specific compounds in five varieties of *Coffea arabica*, each processed differently after harvest.

The concentration of trigonelline in raw coffee beans varied between 13.38–15.24 mg g^–1^ (Table 8), while in roasted beans, it was between 10.68–12.75 mg g^–1^, values similar to those found by Mehari.^21^ Trigonelline varies between 10 and 22 mg g^–1^ in raw beans and is generally 10 mg g^–1^ or less in roasted beans.^32^ The Tukey test revealed significant variation in trigonelline content among the roasted coffee samples, with Yellow Catuai and Guesha exhibiting the highest concentrations. In the raw beans, Yellow Catuai and Arara stood out with lower trigonelline levels compared with the other cultivars.

**Table 8:** Concentration of Trigonelline, 5-Caffeoylquinic Acid, and Caffeine in Roasted Coffee Samples Using the Proposed Analytical Method with the Respective Tukey Test Results^a^ ^,^ ^b^

	Yellow Catuai	Guesha	Red Catuai	Arara	Laurina
	Trigonelline (mg g^–1^)				
roasted	12.654 Ba	12.764 Ba	10.894 Bb	10.680 Bb	10.966 Bb
raw	13.781Ab	15.014 Aa	15.239 Aa	13.386 Ab	15.394 Aa
degradation rate	8.17%	14.98%	28.51%	20.22%	28.76%
	5-Cafeoylquinic acid (mg g^–1^)				
roasted	25.801 Bab	26.867 Ba	20.397 Bc	20.507 Bc	21.535 Bbc
raw	58.282 Aa	57.885 Aa	49.414 Ab	54.818 Aa	54.465 Aa
degradation rate	55.73%	53.58%	58,72%	62.59%	60.46%
	Caffeine (mg g^–1^)				
roasted	12.328 Ba	10.602 Bb	12.212 Aa	12.564 Aa	7.934 Ac
raw	12.724 Aa	12.547 Aab	12.618 Bb	12.594 Aab	7.965 Ac
degradation rate	3.11%	15.0%	3.21%	0.23%	0.38%

a Values with the same letter do not differ according to the Tukey test (5%).

b Lowercase letters compare the means between cultivars, and uppercase letters compare the means of the raw and roasted processes.

The five varieties were roasted under similar conditions but did not show the same rate of thermal degradation. Yellow Catuai (8.17%) and Guesha (14.98%) showed low degradation, while the other varieties showed higher rates, with Red Catuai having the highest degradation (28.51%). The different degradation rates are attributed to the internal distribution of the compounds and the other chemical profiles of each variety. During roasting, trigonelline is degraded to furfural, niacin, nicotinic acid, and volatile compounds.^33^ The trigonelline can be an important chemical parameter for a quality coffee drink. Other studies show a positive correlation between trigonelline concentration and better beverage quality for both Arabica and Robusta coffees.^34,35^

The concentrations of 5-caffeoylquinic acid for raw coffee beans ranged from 49.41 to 58.28 mg g^–1^ (Table 8). A variation between 60 and 68 mg g^–1^ was found in a study of eight cultivars in Ethiopia and different geographical origins.^21^ Similarly, the interaction between cultivars and processing methods influenced the final 5-ACQ content, with a variation of 40 and 60 mg g^–1^ reported.^36^ These results indicate that the variation in chlorogenic acid is influenced by geographic origin, cultivar, and postharvest method.

Tukey’s test showed that only the average of the raw beans of Red Catuai was significantly lower among the varieties. In roasted beans, the values ranged from 20.39 to 26.87 mg g^–1^ due to hydrolysis during roasting, in which 5-caffeoylquinic acid is hydrolyzed to form quinic acid, caffeic acid, and other compounds.^37^ The degradation rates of 5-caffeoylquinic acid in the different varieties differed during the roasting process. This indicates that the varieties interact differently when exposed to the temperatures required for roasting, forming new acidic compounds and phenolic derivatives and influencing their concentration. The degradation rate in roasted beans was lower in Guesha (53.58%) and Arara (62.59%). Since coffee contains other chlorogenic acids, degradation products may also come from these other acids.^38^ Studies show that the concentration of chlorogenic acids increases when the coffee plant is affected by rust (*Hemileia vastatrix*).^39^ Also, the higher concentration of chlorogenic acid is correlated with fruit maturation stages and cup quality.^34,40^ The method optimized in this study is ideal for future studies of these effects.

Caffeine concentrations ranged from 7.93 to 12.72 mg g^–1^ in raw and roasted coffee beans, similar to those found in the literature.^21^ When comparing raw and roasted beans of the same variety, it was observed that there was a significant variation in concentration only in Guesha beans. The lack of significant variation between the other four varieties is because caffeine belongs to a class of compounds called methylxanthines, which is considered a natural ergogenic that is not degraded or hydrolyzed during the roasting process.^41^ Degradation was significant only for the Guesha variety (15%) and may be related to the roasting and processing processes to which the beans were subjected. The method proved to be efficient in showing that the Laurina genotype has a lower caffeine content, as reported in the literature.

Recent studies, such as those by Santanatoglia et al. and Farag et al., confirm the reliability of LC-MS/MS for the simultaneous determination of caffeine, trigonelline, and chlorogenic acids, serving as a comparative basis for validating more accessible methods.^42,43^ The good agreement between the values obtained by the proposed methodology and the LC-MS/MS data reinforces its applicability as a viable alternative for quality control and phytochemical analysis laboratories, especially in contexts where access to highly complex techniques is limited. The proposed methodology can be compared with established reference methods, such as ISO 20481:2008 and AOAC 979.08, which establish chromatographic parameters for the quantification of caffeine by HPLC-UV in coffee matrices.^44,45^ These methods utilize a mobile phase with a higher proportion of organic solvents and more elaborate sample preparation steps (such as reflux extraction). The methodology proposed in this work offers shorter analysis time and lower environmental impact, representing a cleaner, safer, and faster analytical alternative suitable for industrial quality control and laboratory studies.

In conclusion, the method developed for the simultaneous analysis of trigonelline, 5-caffeoylquinic acid, and caffeine in extracts of raw and roasted coffee using HPLC-UV has demonstrated effectiveness in terms of linearity, accuracy, and precision for the concentrations tested. This method offers a rapid and efficient alternative for analyzing the compositions of these bioactive compounds in coffee samples. The concentrations of trigonelline, 5-caffeoylquinic acid, and caffeine vary significantly among different coffee samples. However, the most important variation is observed in 5-caffeoylquinic acid. This compound may be essential for understanding how the composition of chlorogenic acids affects the characteristics of different coffees.

## Supplementary Material



## Notes

Tables with statistical test results for linearity (ANOVA and *t* test for coefficients), figures showing the effects of Plackett–Burman design on retention time and peak area, comparison of analytical curves with and without analyte standard addition (doping) in green and roasted coffee extracts for trigonelline, 5-caffeoylquinic acid, and caffeine (PDF)
