# Supplementary material for: Optimization of Methodology for Simultaneous Quantification of Trigonelline, 5‑Caffeoylquinic Acid, and Caffeine in Green and Roasted Coffee Extracts by HPLC
Source: ACS Omega. 2025 Aug 25;10(35):40304–12. doi: 10.1021/acsomega.5c05526 (PMC12423835; doi:10.1021/acsomega.5c05526)
Supplement: Supplementary file 1 [file ao5c05526_si_001.pdf]

Supplementary material

Optimization of methodology for simultaneous quantification of trigonelline, 5-caffeoylquinic acid, and caffeine in green and roasted coffee extracts by HPLC

Walace Breno da Silva[1*], Larissa Martins Rocha[1], Lucca Dornelas Guimarães Moura[2], Márcio Santos Soares[3], Sabrina Alves da Silva[4], Daniele Birck Moreira[5], Pedro Ivo Vieira Good God[2] and Geraldo Humberto Silva[1]*

[1]Institute of Exact and Technological Sciences, Laboratory of Development of Natural Agrochemicals, Federal University of Viçosa, Highway MG-230, Km 7 - Rural Area, Rio Paranaíba, 38810-000, MG, Brazil

[2]Institute of Agrarian Sciences, Laboratory of Biochemistry and Molecular Genetics, Federal University of Viçosa, Highway MG-230, Km 7 - Rural Area, Rio Paranaíba 38810-000, MG, Brazil

[3]Institute of Exact and Technological Sciences, Laboratory of Environmental Geochemistry and Natural Products, Federal University of Viçosa, Highway MG-230, Km 7 - Rural Area, Rio Paranaíba 38810-000, MG, Brazil

[4]Institute of Animal Health and Production, Federal Rural University of the Amazon, Avenue Presidente Tancredo Neves, N° 2501, Terra Firme, Belém 66077-830, PA, Brazil

[5]Laboratory of Molecular Genetics of Plants and Phytopathogens, Mato Grosso State University Pro-Central-West Network, Highway MT-358, Km 7, Jardim Aeroporto, Tangará da Serra 78301-532, MT, Brazil

***Corresponding authors**

Walace Breno da Silva - Institute of Chemistry, Department of Biochemistry and Organic Chemistry, São Paulo State University (UNESP), Araraquara 14800-060, SP, Brazil; Email: walace.silva@unesp.br

Table S1. The results of the F-test of the analysis of variance of the linearity test.

	F-statistic	*p*-value
Trigonelline	86741.22	< 0.05
5-Caffeoylquinic acid	56743.12	< 0.05
Caffeine	67894.21	< 0.05

Table S2. The results of the t-test for the linear and angular coefficients of the straight-line equation.

Coefficients	t-statistic	*p*-value
	Trigonelline	
Linear	7.164484	2.25×10^{-6}
Angular	158.2968	5.40×10^{-27}
	5-Caffeoylquinic acid	
Linear	-2.58	$1.23 \text{ x } 10^{-2}$
Angular	62.4339	$1.53 \text{ x } 10^{-20}$
	Caffeine	
Linear	3.78	$2.0 \text{ x } 10^{-4}$
Angular	191.1166	$2.65 \text{ x } 10^{-28}$

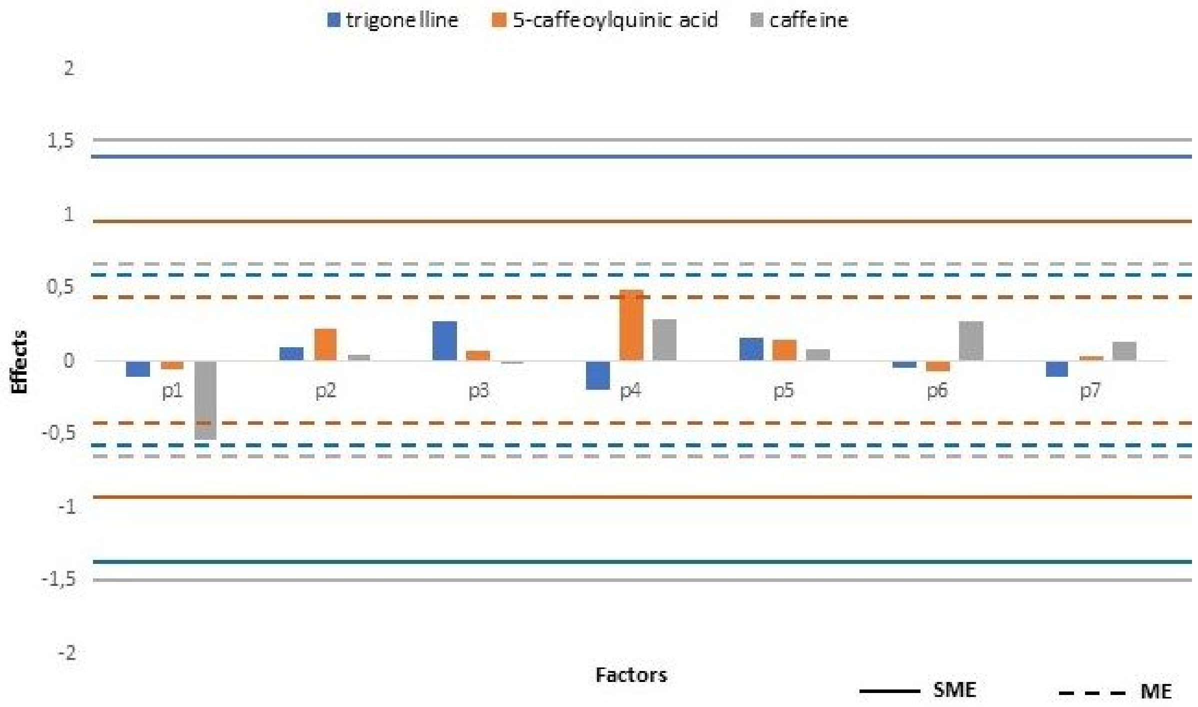

Figure S1. The effects of Plackett–Burman variations on retention time. Orange refers to ferulic acid and blue to caffeic acid. The dashed lines refer to the margin of error (ME) and the continuous lines to the simultaneous margin of error (SME). (p1) Acetonitrile concentration in the mobile phase; (p2) column temperature (°C); (p3) mobile phase flow rate (mL min^{-1}); (p4) wavelength (nm); (p5) acetonitrile brand; (p6) acid concentration in the mobile phase; (p7) acid type.

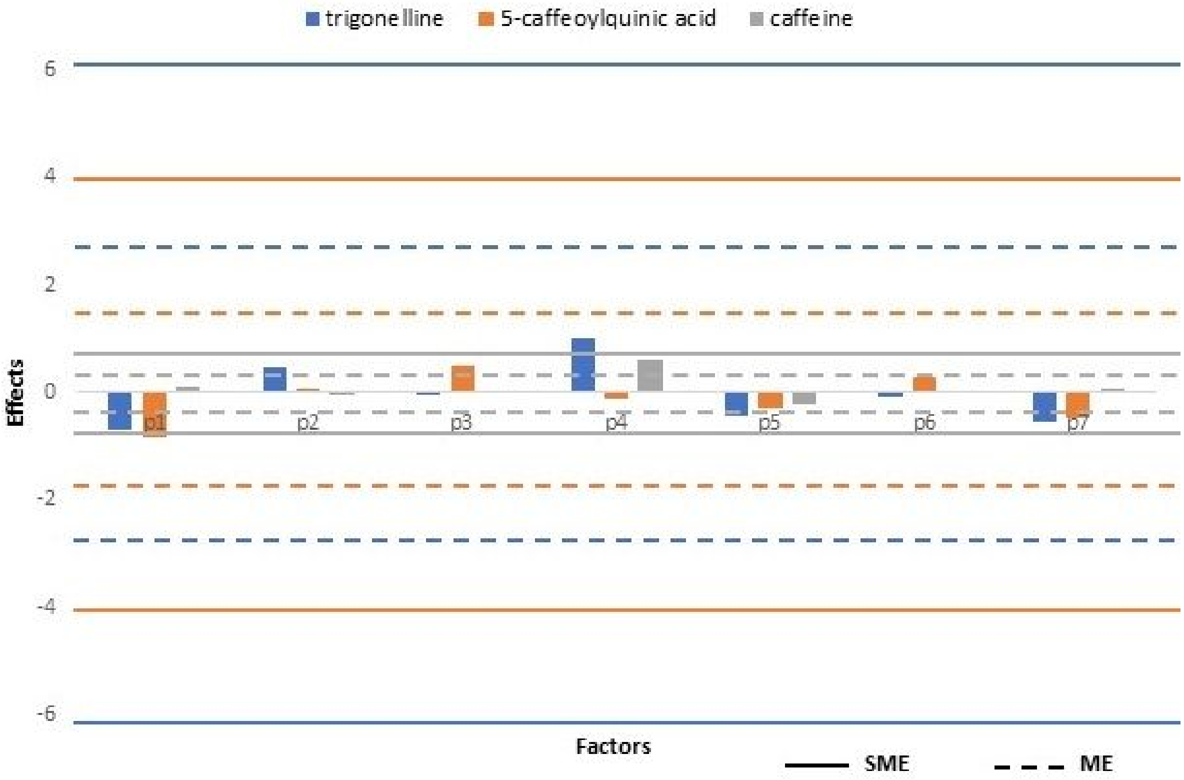

Figure S2. The effects of variations using the Plackett–Burman design in relation to area. Orange refers to ferulic acid and blue to caffeic acid. The dashed lines refer to the margin of error (ME), and the continuous lines refer to the simultaneous margin of error (SME). (p1) Acetonitrile concentration in the mobile phase; (p2) column temperature (°C); (p3) mobile phase flow rate (mL min^{-1}); (p4) wavelength (nm); (p5) acetonitrile brand; (p6) acid concentration in the mobile phase; (p7) acid type.

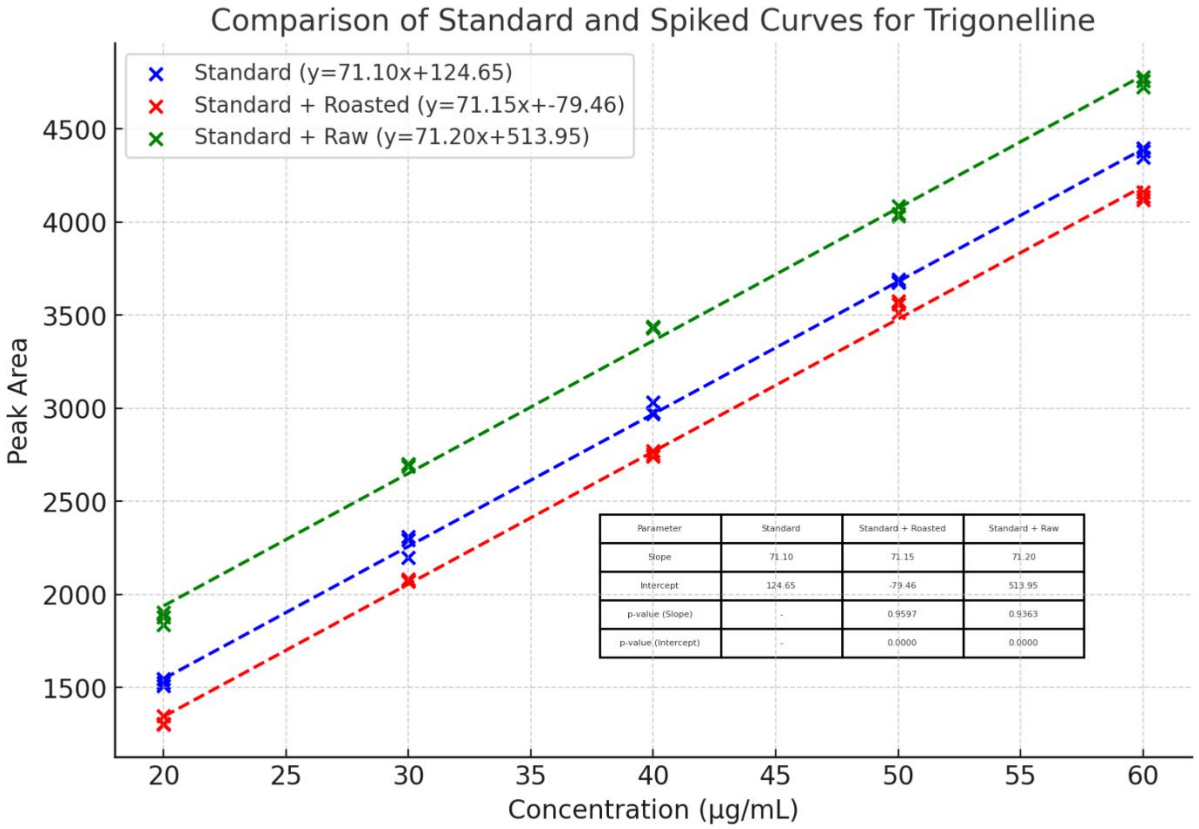

Figure S3. Comparison between analytical curves with trigonelline (blue lines) and with the addition of analyte standard (doping), in raw/green (green lines) and roasted (red lines) coffee extracts, at concentrations of 20.0, 30.0, 40.0, 50.0 and 60.0 μg mL^{-1}.

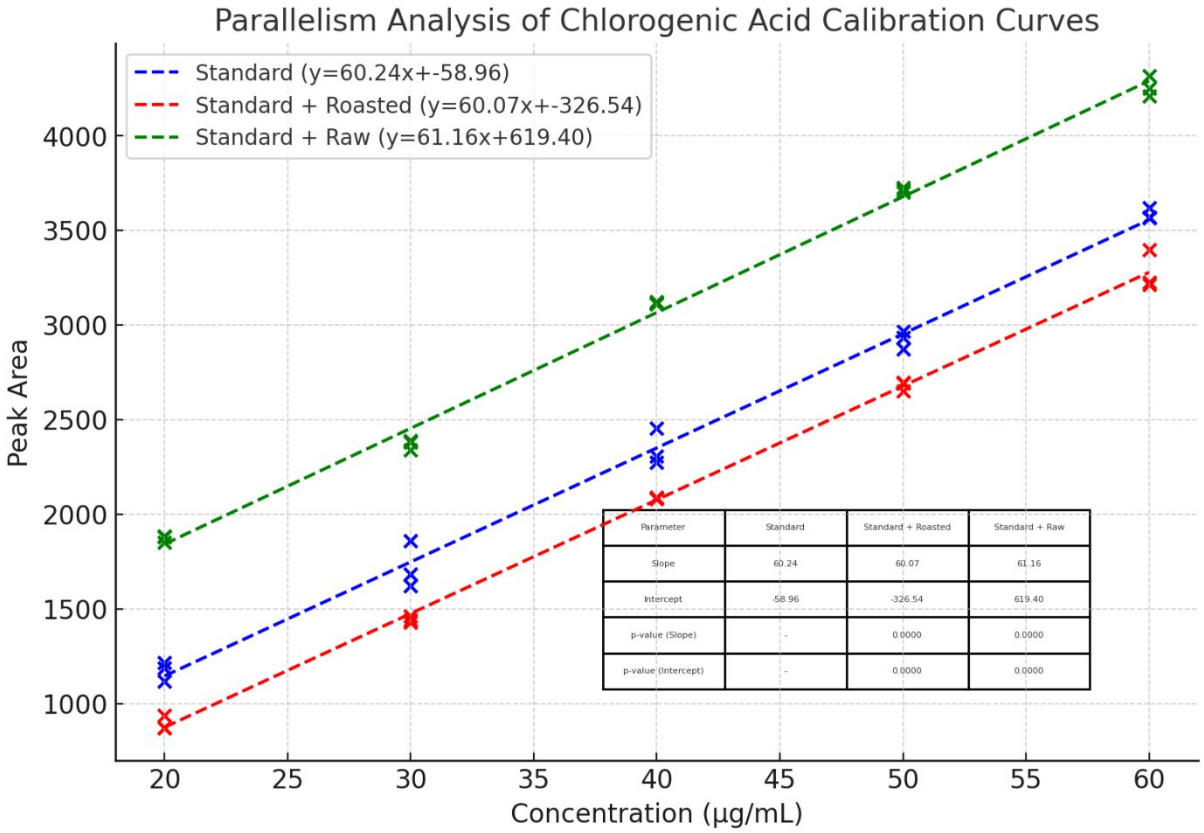

Parameter	Standard	Standard + Roasted	Standard + Raw
Slope	60.24	60.07	61.16
Intercept	-58.96	-326.54	619.40
p-value (Slope)	-	0.0000	0.0000
p-value (Intercept)	-	0.0000	0.0000

Figure S4. Comparison between analytical curves with 5-Cafeoylquinic acid (blue lines) and with the addition of analyte standard (doping), in raw/green (green lines) and roasted (red lines) coffee extracts, at concentrations of 20.0, 30.0, 40.0, 50.0 and 60.0 µg mL^{-1}.

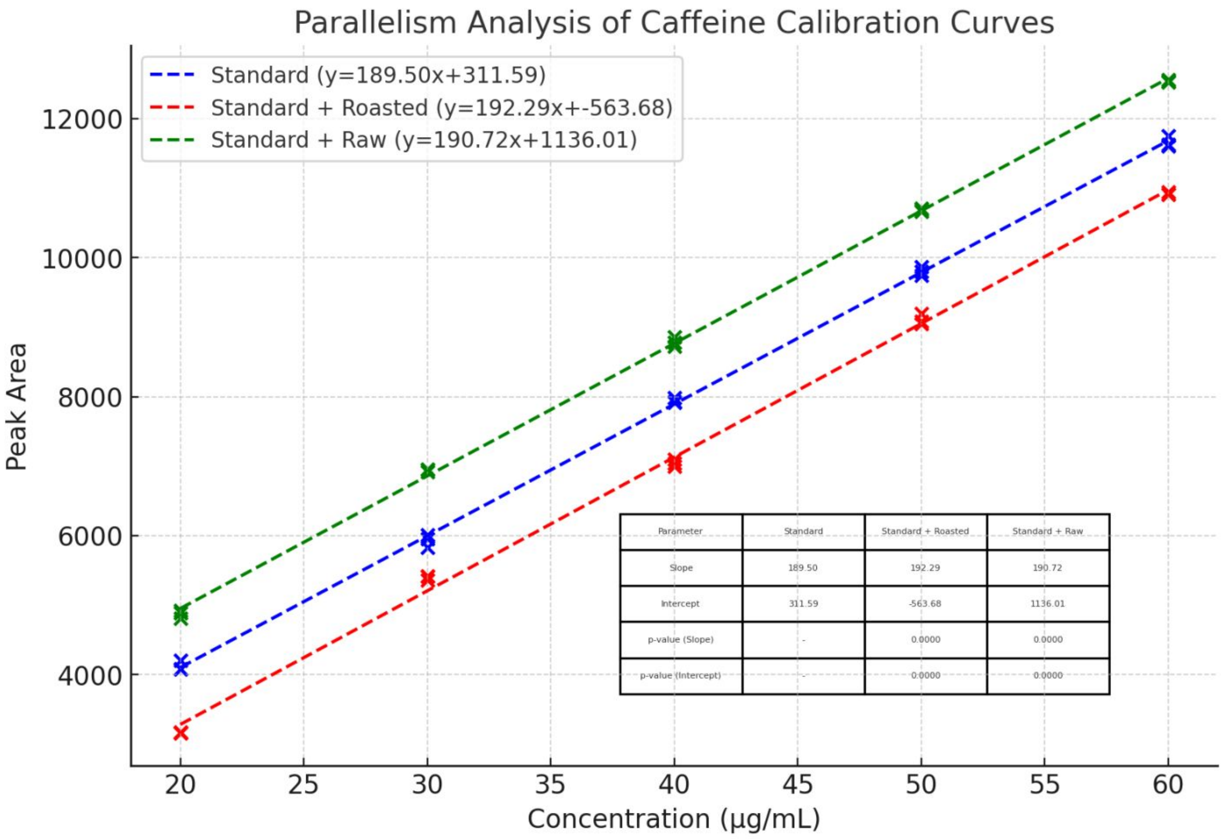

Parameter	Standard	Standard + Roasted	Standard + Raw
Slope	189.50	192.29	190.72
Intercept	311.59	-563.68	1136.01
p-value (Slope)	-	0.0000	0.0000
p-value (Intercept)	-	0.0000	0.0000

Figure S5. Comparison between analytical curves with Caffeine (blue lines) and with the addition of analyte standard (doping), in raw/green (green lines) and roasted (red lines) coffee extracts, at concentrations of 20.0, 30.0, 40.0, 50.0 and 60.0 μg mL^{-1}.
